# The antinuclear antibody HEp-2 indirect immunofluorescence assay: a survey of laboratory performance, pattern recognition and interpretation

**DOI:** 10.1186/s13317-020-00146-w

**Published:** 2021-02-27

**Authors:** Anne E. Tebo, Robert L. Schmidt, Kamran Kadkhoda, Lisa K. Peterson, Edward K. L. Chan, Marvin J. Fritzler, Mark H. Wener

**Affiliations:** 1 Department of Pathology, University of Utah, Salt Lake City, UT USA; 2 ARUP Institute for Clinical and Experimental Pathology, Salt Lake City, UT USA; 3 Immunopathology Laboratory, Robert J. Tomsich Pathology & Laboratory Medicine Institute, Cleveland Clinic, Cleveland, OH USA; 4 Department of Oral Biology, University of Florida, Gainesville, FL USA; 5 Department of Medicine, Cumming School of Medicine, University of Calgary, Calgary, AB Canada; 6 Department of Laboratory Medicine and Pathology & Department of Medicine, University of Washington, Seattle, WA USA

**Keywords:** Anti-nuclear antibodies, Cytoplasmic patterns, Performance survey, Indirect immunofluorescence, Mitotic patterns, Nuclear patterns

## Abstract

**Background:**

To evaluate the interpretation and reporting of antinuclear antibodies (ANA) by indirect immunofluorescence assay (IFA) using HEp-2 substrates based on common practice and guidance by the International Consensus on ANA patterns (ICAP).

**Method:**

Participants included two groups [16 clinical laboratories (CL) and 8 in vitro diagnostic manufacturers (IVD)] recruited via an email sent to the Association of Medical Laboratory Immunologists (AMLI) membership. Twelve (n = 12) pre-qualified specimens were distributed to participants for testing, interpretation and reporting HEp-2 IFA. Results obtained were analyzed for accuracy with the intended and consensus response for three main categorical patterns (nuclear, cytoplasmic and mitotic), common patterns and ICAP report nomenclatures. The distributions of antibody titers of specimens were also compared.

**Results:**

Laboratories differed in the categorical patterns reported; 8 reporting all patterns, 3 reporting only nuclear patterns and 5 reporting nuclear patterns with various combinations of other patterns. For all participants, accuracy with the intended response for the categorical nuclear pattern was excellent at 99% [95% confidence interval (CI): 97–100%] compared to 78% [95% CI 67–88%] for the cytoplasmic, and 93% [95% CI 86%–100%] for mitotic patterns. The accuracy was 13% greater for the common nomenclature [87%, 95% CI 82–90%] compared to the ICAP nomenclature [74%, 95% CI 68–79%] for all participants. Participants reporting all three main categories demonstrated better performances compared to those reporting 2 or less categorical patterns. The average accuracies varied between participant groups, however, with the lowest and most variable performances for cytoplasmic pattern specimens. The reported titers for all specimens varied, with the least variability for nuclear patterns and most titer variability associated with cytoplasmic patterns.

**Conclusions:**

Our study demonstrated significant accuracy for all participants in identifying the categorical nuclear staining as well as traditional pattern assignments for nuclear patterns. However, there was less consistency in reporting cytoplasmic and mitotic patterns, with implications for assigning competencies and training for clinical laboratory personnel.

## Introduction

The presence of antinuclear antibodies (ANA) is a hallmark and classification criterion for a number of systemic autoimmune rheumatic diseases (SARD). ANA testing is usually performed as part of the initial diagnostic workup when suspicion of an underlying autoimmune disorder is high. The indirect immunofluorescence antibody (IFA) technique on HEp-2 substrate has been considered the traditional and preferred method for detecting ANA by some [1]. It allows detection of antibody binding to specific intracellular targets, resulting in diverse staining patterns that are usually categorized based on the cellular components recognized and the degree of binding, as reflected by the fluorescence intensity or titer [2, 3]. As a screening tool, the recognition of a well-defined HEp-2 IFA staining pattern may be helpful in determining the most likely specific autoantibodies present, as well as suggesting possible clinical associations for known specificities [3, 4]. In this regard, a positive HEp-2 IFA screening pattern can guide confirmatory testing and may also be useful for elucidating a specific clinical diagnosis or prognosis. Thus, the provision of HEp-2 IFA patterns and titers is considered to be clinically valuable with favorable utility in comparison with other methods for ANA detection [1–11].

The nuclear IFA staining patterns most commonly recognized and reported by clinical laboratories include homogeneous, speckled, centromere, and nucleolar [1–4, 12–14]. Use of HEp-2 cell substrates, permits detection of additional nuclear staining patterns, as well as reactivity with cell constituents in compartments outside the nucleus (cytoplasmic) and cell components associated with mitosis (mitotic) [2, 4, 12–14]. However, the reactivity and type of autoantigens associated with these patterns may vary among HEp-2 substrates from different manufacturers [15]. Furthermore, the expertise required to identify the different patterns and sub-classify their variants may not be universally available in clinical laboratories. Traditional legacy recommendations for reporting ANA patterns on HEp-2 cells continue to significantly influence clinical laboratory reporting [16, 17].

The first **I**nternational **C**onsensus on **A**ntinuclear Antibody (ANA) **P**atterns (ICAP) was published in 2015 to systematize and update reporting of autoantibody patterns detected by IFA using HEp-2 cell substrates [12]. The goal of this initial and subsequent publications was to optimize usage of HEp-2 IFA patterns in patient care, by promoting standardization, harmonization and understanding of autoantibody test nomenclature and providing guidelines for test interpretation and reporting [4, 12–15, 18]. To-date, 30 HEp-2 IFA nuclear, cytoplasmic and mitotic patterns have been elucidated by ICAP and presented in a classification tree (www.anapatterns.org). The ICAP guidelines indicate that ‘expert-level’ laboratories would report all the HEp-2 IFA patterns, whereas those designated as ‘competent-level’ laboratories would report 6 nuclear and 5 cytoplasmic HEp-2 IFA patterns [12].

In a previously reported survey administered in cooperation with the Association of Medical Laboratory Immunologists (AMLI), a significant number of respondents were unaware of the ICAP initiative, although a majority agreed on the need to standardize the nomenclature and reporting of HEp-2 IFA results [19]. Based on the responses from this survey, a consensus to improve ICAP awareness and further enhance HEp-2 IFA assessment through increased collaboration between ICAP and the clinical laboratory community was suggested with emphasis on education and availability of reference materials. As others have also reported [20, 21], many laboratories around the world are inclined to adopt the ICAP nomenclature and embrace the recommendations provided in these consensus guidelines.

The objective of this study was to evaluate the performance of HEp-2 IFA interpretation based on nuclear, cytoplasmic and mitotic staining in an endeavor to characterize competency as outlined in the ICAP classification.

## Materials and methods

### Participants and recruitment

Participants for the survey were recruited via an email sent to the Association of Medical Laboratory Immunologists (AMLI) membership (a professional organization focused on immunological laboratory testing with 105 active members) and 5 in vitro diagnostics manufacturers (IVD). The requirements to participate included testing pre-defined specimens by IFA using HEp-2 substrate and reporting the patterns observed based on specific “traditional” (as defined below) as well as ICAP (www.anapatterns.org) nomenclature for reading and reporting ANA patterns. Sixteen (n = 16) clinical laboratories (CL) and all 5 in vitro diagnostics manufacturers (IVD) agreed to participate in the study. Three additional IVD (2 in the US and 1 in Europe), contacted the organizers to participate in the survey. Overall, 16 CL and 8 IVD participated in this performance survey (Additional file 1: Table S1).

### Study specimens and survey

Twelve (n = 12) specimens were used in the survey. The specimens were chosen based on an assessment of need (described below) by some of the authors (AET, LKP, EKLC, MJF, MHW). The following attributes were taken into consideration: 1) the three main categorical group of HEp-2 IFA patterns, 2) the ICAP guidance for both competent and expert levels, 3) the clinical significance of patterns and 4) whether or not proficiency testing was available for specific patterns. The authors also wanted to evaluate how participants would interpret nuclear staining associated with anti-topoisomerase I antibodies given recent ICAP guidance for evaluating this complex pattern [22]. The twelve pre-specified HEp-2 IFA-positive specimens labeled ANA-001 through ANA-012 included those positive for nuclear [ANA-002, ANA-003, ANA-005, ANA-006, ANA-007, ANA-010, ANA-011], cytoplasmic [ANA-004, ANA-008, ANA-009] and mitotic [ANA-001, ANA-012] categorical groups of IFA patterns (Table 1). All specimens for the survey and their intended responses were obtained from Plasma Services Group Inc. (PSG: Huntington Valley, PA, USA, https://www.plasmaservicesgroup.com/). Specimens were qualified at PSG using routinely available methods and also verified in the laboratories of one or more expert members of ICAP (PSG, personal communication).

**Table 1:** Survey specimens and their characteristics

Specimen^a^	Cellular staining	Traditional nomenclature	ICAP nomenclature	ICAP level
ANA-001	Mitotic	Mitotic	Spindle fiber, AC-25	Expert
ANA-002	Nuclear	Discrete nuclear dots	Multiple nuclear dots, AC-6	Expert
ANA-003	Nuclear	Speckled	Coarse speckled, AC-5	Expert
ANA-004	Cytoplasmic	Cytoplasmic	Reticular/AMA, AC-21	Competent
ANA-005	Nuclear	Centromere	Centromere, AC-3	Competent
ANA-006	Nuclear	Nucleolar	Homogeneous nucleolar, AC-8	Expert
ANA-007	Nuclear	Speckled	DFS, AC-2	Competent
ANA-008	Cytoplasmic	Cytoplasmic	DFS, AC-19	Expert
ANA-009	Cytoplasmic	Cytoplasmic	Fine speckled, AC-20	Expert
ANA-010	Nuclear	Speckled	Fine speckled, AC-4	Expert
ANA-011	Nuclear	Speckled/Other*	Anti-topoisomerase I, AC-29^b^	Expert
ANA-012	Mitotic	Mitotic	NuMA-like, AC-26^c^	Expert

^a^number, *ICAP* **I**nternational **C**onsensus on **A**ntinuclear Antibody **P**attern, *AC* anti-cell, *AMA* anti-mitochondrial antibodies, *DFS* dense fine speckled, *NuMA* nuclear mitotic apparatus protein. ^b^The AC-29 Anti-topoisomerase pattern I is a compound pattern, classified within ICAP as a speckled pattern. The complex pattern involves speckled nuclear staining, and also includes staining of the condensed chromatin, cytoplasmic staining, staining of the nucleolar organizing region in mitotic cells, and variable nucleolar staining of interphase cells. ^c^For this specimen, AC-25 was also considered acceptable

Survey specimens were shipped to all participants in January 2020 with detailed instructions for testing as well as a report form to record and return results to one of the organizers (AET). Parameters to be recorded by checking the survey form included the three categorical groups of HEp-2 IFA patterns reported (nuclear, cytoplasmic or mitotic); commonly used nomenclature (also referred to as traditional in this investigation) for 5 HEp-2 IFA nuclear patterns (homogeneous, speckled, centromere, nucleolar, discrete nuclear dots), mitotic, cytoplasmic or ‘other’ in accord with legacy classification approaches [16, 17]; and a result based on the ICAP classification tree (www.anapatterns.org), which includes more detailed sub-pattern classification than commonly reported. In addition, the participants were requested to provide information about how the images were read and interpreted (manual and/or automation-assisted reading); the years of experience of the reading technologist(s); the manufacturer of the HEp-2 substrate; the laboratory’s typical practice about reporting only nuclear patterns vs also reporting cytoplasmic and/or mitotic patterns when the ANA test is requested; the screening dilution(s) of serum used for detection of ANA in performing the HEp-2 IFA; and the titer of the ANA, based on serial dilution of the tested specimen. There were two types of participants: clinical laboratories (CL) and in vitro diagnostic manufacturers (IVD). After the results were tabulated, participants were not afforded the opportunity to adjust or revise responses based on the responses of other respondents. Some of the participating CL included those directed by the authors, but the authors did not participate in assigning the patterns reported from their laboratories.

### Data analyses

We compared participants’ HEp-2 IFA pattern classification of specimens against a consensus classification. The primary outcome was the percent accuracy between the participant and consensus classification. We studied the impact of three factors on accuracy: pattern classification hierarchy or nomenclature, participant organization type, and participant experience. We examined three hierarchial nomenclatures: 1) group category (nuclear, cytoplasmic, mitotic); 2) specific traditional pattern descriptions (e.g. speckled, nucleolar, etc.); and 3) sub-pattern classification using the ICAP nomenclature. We refer to these as the group, traditional and ICAP classification methods. Each participant was classified according to the organizational type and the reporting experience at their institution. There were two types of organizations: 1) clinical laboratories (CL) and 2) in vitro diagnostic manufacturers (IVD). Organizations were classified as experienced if they routinely reported all group categories and inexperienced if they did not routinely report all three group categories. Using these three factors and their associated variables, we sought to answer the following questions:

1. Was accuracy associated with the classification method?
2. Was accuracy associated with experience?
3. Was accuracy associated with organization type among experienced participants?
4. Was accuracy associated with categories within nomenclature methods?

We used logistic regression to determine the association between accuracy and the three factors. Outcomes were reported as odds ratios (OR). P-values were adjusted for multiple comparisons using the method of Holm. Statistical analyses were performed using Stata 16.2 (Stata Corp LLP).

## Results

### Characteristics of survey participants

There were 24 participants: 16 were CL (13 in the United States and 3 in Canada) and 8 IVD. Most of the CL used kits from three main IVD that also participated in the survey (Table 2). The majority of the CL read, interpreted and determined HEp-2 IFA patterns and titers manually using 1:40 as cut-off for HEp-2 IFA determinations. The median years of experience for technologists who participated in the survey was 10 years for CL compared to 20 years for the IVD participants.

**Table 2:** Characteristics of survey participants

Characteristics		CL, N (%)^a^	IVD, N (%)^a^
HEp-2 kit	Bio-Rad	4 (25.00)	See legend
	Euroimmun	5 (31.25)	
	Inova	6 (37.50)	
	MBL Bion	1 (6.25)	
Type of reader	Manual only	9 (56.25)	5 (62.50)
	Manual and Automated	7 (43.75)	3 (37.25)
Cut-off (titer)	< 1:10	1 (6.25)	0 (00.00)
	< 1:40	12 (75.00)	6 (66.67)
	< 1:80	3 (18.75)	2 (22.22)
	< 1:100	0 (00.00)	1 (11.11)
Technologist experience^b^	1–5 years	7 (33.30)	0 (00.00)
	6–10 years	4 (12.10)	1 (11.11)
	> 10 years	10 (47.60)	8 (88.89)
	Median (range), years	10 (1-45)	20 (2-51)
Patterns reported	Nuclear only	3 (18.75)	0 (00.00)
	^c^Cytoplasmic and nuclear	2 (12.50)	0 (00.00)
	Mitotic and nuclear	3 (18.75)	0 (00.00)
	^c^All patterns	8 (50.00)	8 (100.00)

^a^Number (N) of clinical laboratories (CL) or in vitro diagnostic manufacturers (IVD) unless otherwise stated. Participating IVD manufacturers included: AESKU Diagnostics, Bio-Rad, Euroimmun, Inova, ImmunoConcepts, Scimedx, ThermoFisher and Zeus. CL labs using Inova HEp-2 substrate kits use < 1:40 or < 1:80 as cut-off for ANA determinations. ^b^More than one technologist was involved in the reading and interpretation of the results in some laboratories. ^c^One laboratory in each group reports cytoplasmic pattern only as a comment

The number of categorical groups of patterns typically reported by the CL was variable. Among the 16 CL, 3 indicated they reported only nuclear patterns, 3 indicated they reported nuclear and mitotic patterns, 2 reported nuclear and cytoplasmic patterns (with one of the 2 reporting the cytoplasmic pattern as a comment, not as a ‘positive ANA’) and 8 indicated the laboratory reported nuclear, cytoplasmic and mitotic patterns (with one of the 8 reporting the cytoplasmic patterns as a comment, not as a ‘positive ANA’). Among the 8 IVD, 6 indicated they report patterns in all 3 categorical groups, however, all provided responses to all categories in the survey.

### Performance of participants based on the categorical HEp-2 IFA groups

The accuracy for reporting the nuclear pattern was 99% (95% CI 95–100%) for all participants, while the cytoplasmic and mitotic group categories had accuracy of 78% (CI 66–87%) and 93% (CI 81–88%), respectively (Table 3). The overall accuracy of IVD was greater than accuracy of CL (97% vs 91%, Additional file 2: Table S2a) in assigning the HEp-2 IFA group categories of patterns of all specimens. This difference was statistically significant (p = 0.04). Combined, the two organization types (CL and IVD) had an overall accuracy of 93% (95% CI 89–96%) for determining the three categorical groups of HEp-2 IFA patterns.

**Table 3:** Performance of Participants in the Three HEp-2 IFA Group Categories

Group category	Specimens (Number, n)	Observations (Number, n)	Accuracy (95% CI)
Nuclear	7	168	99 (95–100)
Cytoplasmic	3	63	78 (66–87)
Mitotic	2	45	93 (81–98)
Overall	12	276	93 (89–96)

*CI* confidence interval

### Performance of participants based on “traditional” and ICAP nomenclatures

Participants were asked to report results based on survey-suggested classifications (referred here as “traditional”) as well as the ICAP nomenclature. The overall accuracy for the traditional nomenclature system was 87% (95% CI 82–90%), Table 4. Only the specimen (ANA-011) with antibodies to DNA topoisomerase I was reported with accuracy less than 80% and that complex mixed/compound pattern had not been included in many ANA pattern classification teaching schemes prior to its recent inclusion as a distinct ICAP pattern [20]. Among the traditional pattern reports, two specimens (ANA-003, centromere and ANA-006, nucleolar) were reported with accuracy of 100%.

**Table 4:** Performance of participants in the traditional and ICAP nomenclature systems

Specimen ID	Traditional	Observations (n)	Accuracy (95% CI)	ICAP	Observations (n)	Accuracy (95% CI)
ANA-005	Centromere	24	100 (86–100)	Centromere, AC-3	20	100 (83–100)
ANA-002	DND	24	96 (73–99)	MND, AC-6	20	85 (60–95)
ANA-003	Speckled	24	100 (86–100)	Nuclear CS, AC-5	18	67 (41–85)
ANA-007	Speckled	24	96 (73–99)	Nuclear DFS, AC-2	20	80 (55–93)
ANA-010	Speckled	24	83 (62–94)	Nuclear FS, AC-4	19	37 (18–61)
ANA-011	Speckled/Other	24	42 (23–63)	Anti-topo I, AC-29	21	62 (39–81)
ANA-006	Nucleolar	24	100 (86–100)	Homo nucleolar, AC-8	20	70 (46–87)
ANA-004	Cytoplasmic	19	100 (86–100)	AMA, AC-21	19	89 (66–97)
ANA-008	Cytoplasmic	20	90 (65–98)	Cytoplasmic DFS, AC-19	19	79 (53–92)
ANA-009	Cytoplasmic	19	53 (30–74)	Cytoplasmic FS, AC-20	19	42 (22–66)
ANA-001	Mitotic	22	86 (64–96)	Spindle fiber, AC-25	20	75 (50–90)
ANA-012	Mitotic	24	92 (70–98)	NuMA-like, AC-26	22	95 (71–99)
	Overall	250	87 (82–90)	Centromere, AC-3	237	74 (68–79)

*ICAP* **I**nternational **C**onsensus on **A**ntinuclear **A**ntibody **P**atterns). *ID* identification number, *AC* anti-cell, *DND* discrete nuclear dots, *MND* multiple nuclear dots, *CS* coarse speckled, *DFS* dense fine speckled, *FS* fine speckled, *AMA* anti-mitochondrial antibodies, *homo* homogeneous, *anti-topo I* anti-DNA topoisomerase I, *CI* confidence interval, *NuMA* nuclear mitotic apparatus. Variation in observation numbers in table reflects the fact that some laboratories did not report all cytoplasmic or mitotic categorical group patterns or ICAP patterns for some specimens

The overall accuracy for the ICAP nomenclature reporting system was 74% (95% CI 68–79%), Table 4. For the ICAP nomenclature, 5 out of the 12 (41.7%) specimens were reported with overall accuracy over 80%. These included AC-3: centromere, AC-6: multiple nuclear dots, AC-2: dense fine speckled (with 100% of IVD and 67% of responding CL accurately), AC-21: AMA and AC-26: NuMA-like. For ANA-012, participants reporting AC-25 and AC-26 were graded as having consensus for the intended report.

Several specimens yielded unexpected results. For example, ANA-010 was intended to represent a nuclear fine speckled pattern (AC-4), but that specimen was reported as ICAP pattern AC-4 by only a minority of CL and IVD. The survey showed that all participants reported this specimen as having a speckled nuclear pattern using traditional descriptions, but a majority (75% of IVDs and 55% of CL) reported it as having coarse speckled nuclear staining (AC-5) rather than the expected AC-4. Review of images from several participating laboratories revealed that the specimen produced patterns ranging from typical fine speckled to coarse speckled nuclear staining using different HEp-2 cell sources. Similarly, the specimen (ANA-009) intended to represent a cytoplasmic fine speckled pattern (AC-20) was reported with other patterns by a majority of participants and review of images from different laboratories showed a variable pattern of staining depending on the source of the HEp-2 substrate (data not shown).

The specimen with antibodies to topoisomerase I (topo-1, AC-29 pattern) was reported as homogeneous by most (56%) CL participants and as a mixed (homogeneous and nucleolar) pattern by an additional 19% of CL using the common pattern descriptions. The IVD participants reported it as having a variety of mixed nucleolar patterns. Using ICAP nomenclature, 88% of IVD participants and 54% of responding CL participants assigned the specimen as having the AC-29 (anti-topoisomerase I) pattern.

Overall, for all participants, the accuracy was 13% greater for the traditional nomenclature (87%, 95% CI 82–90%) compared to the ICAP nomenclature (74%, 95% CI 68–79%, Additional file 2: Table S2b and Tables 3). However, the accuracy for reporting the ICAP nomenclatures were lower for CL than IVD with an overall accuracy of 81% (95% CI 77–84%, Additional file 2: Table S2). The accuracy of classification was associated with participant type ($\chi_{12}^{2}$ p < 0.0005) and nomenclature system (p < 0.0005). The accuracy of the CL group was 15% less than the IVD group.

To assess the performance of each organization type based on the accuracies for the main categorical, traditional and ICAP nomenclature determinations, the data were stratified, and frequencies of the correct intended responses estimated (data not shown). Both groups were effective in determining the intended nuclear staining, however, the CL group demonstrated lower frequencies of the expected responses for the different nomenclatures. This was most pronounced for the ICAP nomenclature.

### Impact of nomenclature and participant experience on accuracy

Participants had the highest accuracy using the group category nomenclature (Table 3). The average accuracy associated with the group category nomenclature was 93% (95% CI 90–96%). For the traditional nomenclature, the average was 87% (95% CI 83–91%) which was significantly less accurate than the group category nomenclature (OR = 0.48, p = 0.014). The average accuracy of the ICAP nomenclature was 74% (95% CI 68–79%) which was significantly less than the group category nomenclature (OR = 0.20, p = 0.002) and the traditional nomenclature (OR = 0.42, p = 0.002), Table 5. Experienced participants had higher accuracy than nonexperienced participants (OR = 2.2, p < 0.0005). The accuracy of experienced participants was greater than the accuracy of nonexperienced participants for all nomenclatures. The difference was 6% for the group method, 10% for the traditional nomenclature and 13% for the ICAP nomenclature.

**Table 5:** Accuracy of classification based on experience and participant type

Classification nomenclature	Experience	Observations (n)	Accuracy (95% CI)	Average (95% CI)	*P* value
Group category (n = 276)	No	87	89 (82–95)	93 (90–96)	Base
	Yes	189	95 (92–98)		
Traditional (n = 272)	No	84	80 (71–89)	87 (83–91)	0.014
	Yes	188	90 (86–94)		
ICAP (n = 232)	No	67	64 (53–76)	74 (68–79)	0.002^a^ 0.002^b^
	Yes	170	77 (73–84)		
All (n = 785*)*	No	238	79 (73–84)	85 (83–88)	0.002**
	Yes	547	88 (85–91)		

Group category (n = 189)	IVD	96	97 (93–100)	95 (92–98)	Base
	CL*	93	94 (88–99)		
Traditional (n = 188)	IVD	96	97 (93–100)	90 (86–94)	0.05
	CL*	92	83 (75–91)		
ICAP (n = 170)	IVD	95	83 (76–91)	78 (71–84)	0.002^a^ 0.002^b^
	CL*	75	71 (60–81)		
All (n = 547)	IVD	287	92 (89–95)	88 (85–91)	0.002**
	CL*	260	83 (78–88)		

HEp-2 cell IFA patterns were evaluated based on experience for all participants (yes or no), and experienced participant types (in vitro diagnostics manufacturers, IVD) and experienced clinical laboratories (CL*). Experienced CL defined as reporting all 3 main nomenclature categories. All IVD participants reported the three nomenclature categories and are rated experienced. CI: confidence interval, *n* number. ^a^ICAP vs Group category and ^b^Traditional vs ICAP

**Indicates significant difference between groups

### Impact of participant type

All IVD participants were experienced and 8 of the 16 CL participants were experienced (with experience defined as routinely reporting all group categories). Among experienced participants, IVD had greater accuracy than CL (OR = 2.8, p = 0.002, Table 5). On average, the accuracy of the IVD participants was 92% (95% CI 89–95%) and the accuracy of the experienced CL participants was 83% (95% CI 78–88%). The accuracy was associated with nomenclature. The accuracy of the ICAP nomenclature was 78% (95% CI 71–84%) which was significantly lower (OR = 0.16, p = 0.002) than the accuracy of the group nomenclature (95%, 95% CI 92–98%) and significantly lower (OR = 0.35, p = 0.002) than the traditional nomenclature (90%, 95% CI 86–94%). Among the CLs, use of automation-assisted reading trended toward improved accuracy of pattern reporting for both traditional (87% vs 82% accuracy) and ICAP (70% vs 55% accuracy), but these differences were not statistically significant.

### Frequency distribution of end-point titers

**Fig. 1:**
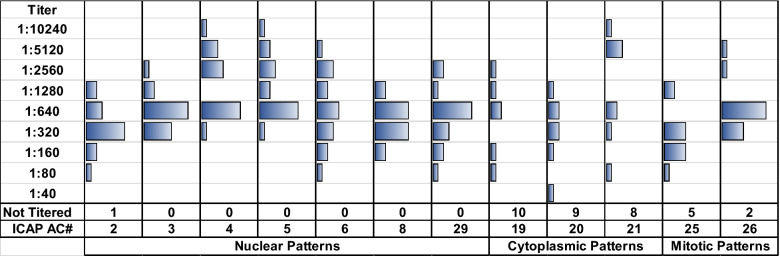
Distribution of end-point titers for survey specimens reported by clinical laboratory (CL) participants. The frequency distributions of titer values for the 12 samples as reported by 16 CL participants is graphically illustrated. The number of CL reporting titer (1:40 to 1:10,240) for each AC-numbered specimen is shown, as well as the number of clinical labs that did not titer the specimen. The distance between vertical lines represents 10 participants

For each of the 12 specimens analyzed, the distribution of the reported antibody titer was recorded. The screening titer of determinations ranged from 1:10 to 1:80. The titers were generally quite variable (Fig. 1, shown for CL). The specimens with cytoplasmic patterns were often not titered, particularly by laboratories that did not routinely report cytoplasmic patterns. Of these specimens, the titer variability was most pronounced for ANA-004 (AMA, AC-21) with positive results demonstrating a bimodal response which spanned eight twofold titers ranging from 1:80 1:10,240 for CL reporting this pattern. Nuclear pattern staining titers also varied substantially, spanning from four to seven twofold titers in different specimens.

## Discussion

Using pre-tested and selected patient serum specimens, we report here the performance of 24 CL and IVD participants recruited from a professional organization focused on immunological laboratory testing and accustomed to the interpretation and reporting of HEp-2 IFA patterns. The specimens included examples from all 3 main categorical patterns (nuclear, cytoplasmic, or mitotic), were reported using ‘traditional’ and ICAP nomenclatures, and included patterns designated by ICAP as associated with both ‘competent’ and ‘expert’ laboratories. Our data demonstrates competence for participants in identifying and reporting common nuclear ANA patterns, but inconsistency in the decision to report and pattern reporting of cytoplasmic and mitotic patterns.

In recent years, efforts to standardize interpretation and reporting of HEp-2 patterns have led to a consensus nomenclature presented by ICAP, a group of experts [4, 12–14] with the purpose of systematic reporting and optimizing the usage of HEp-2 IFA patterns in patient care [4]. In a previous study, we identified increasing awareness of this guidance; availability of reference materials for training and collaboration between professional organizations, IVD and CL amongst others as key elements necessary for improved harmonization of the HEp-2 IFA reporting [19].

In addition to accurately reporting binding of autoantibodies to defined cellular components, the survey also evaluated responses based on “traditional” categorization for nuclear patterns as well as the emerging ICAP nomenclature. As expected, all participants performed better with the more widely used or common traditional HEp-2 IFA nomenclature, which has more emphasis on limited nuclear staining features than required to correctly assign ICAP patterns. While the reason for this could be due to limited familiarity with ICAP, based on the data, other reasons for this can be inferred. First, the “traditional” categorization which can also be referred to as the ICAP *“competent*-*level”* is broad and minimizes the use of fine details and/or integrated pattern recognition in its interpretation. For example, most responders were capable of identifying ANA-003 and ANA-007 as nuclear speckled patterns but failed to accurately demonstrate the intended ICAP nomenclatures, coarse speckled/AC-5, and dense fine speckled/AC-2, respectively. In fact, a number of respondents classified the AC-2 DFS specimen as a mixed nuclear homogeneous and nuclear speckled pattern, as might be expected for traditional classification based on speckled staining of the nucleoplasm and intense chromatin staining. The combination requires integration to assign the nuclear DFS AC-2 ICAP pattern, rather than describing mixed homogeneous and speckled staining pattern with which it might be confused.

The specimen with antibodies to DNA topoisomerase I and the AC-29 staining pattern also demonstrated remarkable challenges of consistent ANA pattern reporting. Under ICAP, AC-29 is considered a sub-pattern of nuclear speckled staining [22], but only a minority of participants reported it as a nuclear speckled pattern using traditional nomenclature. Using “traditional” classifications, the pattern is a compound, mixed staining pattern in which the speckled component may not be perceived as dominant, even though it is consistently observed. In addition to the speckled nuclear staining, there is also staining of condensed chromatin in the mitotic cells, making it difficult to distinguish from homogeneous nuclear staining, as reported by majority of the CL, and nucleolar staining also is often present. Dellavance and colleagues [23] first reported on a composite of five unique HEp-2 staining attributes associated with positivity for anti-topoisomerase I which may not be consistently observed in all HEp-2 substrates and/or serum dilutions [22]. Among the value of the ICAP classification scheme is that interpretation of complex mixed staining may be better reported as a single unifying pattern. In support of this, laboratories accustomed to ICAP classification correctly reported the ICAP AC-29 topoisomerase pattern when asked to use the ICAP nomenclature, although they may not have reported it as a speckled ANA using traditional descriptions.

A recent multicenter analysis to evaluate the interpretation of HEp-2 IFA reported significant differences among laboratories in terms of qualitative results, patterns, and titers, particularly at low levels and in those with speckled patterns [24]. HEp-2 IFA titer determinations have been reported to have clinical significance in predicting risk for disease (healthy vs. disease) as well as association with specific autoantibodies [25–29]. Our data confirm previous reports that ANA titers as reported by individual laboratories vary considerably, and point out another opportunity for harmonization of ANA reporting. With respect to the ICAP nomenclature, our data demonstrated clusters of participants based on the HEp-2 patterns reported by the participants. First, the majority of participants in this survey reliably read and interpreted the centromere, multiple nuclear dots, nuclear dense fine speckled, AMA and NuMA-like sub-patterns. The NuMA-like pattern is considered uncommon, and expected to be recognized by “Expert” level laboratories, but it has a characteristic appearance, and has clinically significant associations with a number of SARD [30]. Second, a significant group of participants could identify challenging ICAP-designated sub-patterns. These include the homogeneous nucleolar, cytoplasmic dense fine speckled, spindle fiber, nuclear coarse speckled, and anti-topoisomerase I patterns. Except for the AMA pattern, the overall performance of the CL participants for specimens with the cytoplasmic patterns was more variable, and lower than the IVD group. These observations have implications for defining competency for CL for cytoplasmic and mitotic patterns.

A minority of participants interpreted the nuclear fine speckled and cytoplasmic fine speckled sub-pattern specimens as intended. The data suggested that the HEp-2 patterns generated by those specimens had a sufficiently variable appearance, based on the kit manufacturer, and/or kit lot, to lead the specimens to appear as different ICAP categories in the hands of different participants. That hypothesis was confirmed by our direct review of the appearance from different laboratories (data not shown). The observations reinforce the need for harmonization of reagents, as well an enhanced training in pattern interpretation, in order to generate consistent results.

Analyses of the performance of the participants showed that the average accuracy with the expected patterns varied based on the hierarchical nomenclature categories and rater groups (CL vs IVD). Combined, both group of participants exceeded 80% average accuracy for two (nuclear and mitotic) of three group categorical patterns. The performance for both groups was more variable based on traditional and ICAP nomenclatures. However, the CL group had more varied average accuracy for both the traditional and ICAP nomenclatures with the ICAP nomenclature demonstrating significantly lower performnace. This may reflect how HEp-2 IFA patterns are reported in the CL and/or the experience of these participants. Notably, the participants in the IVD group had more years of experience than those on the CL group. Furthermore, only half of the CL participants routinely reported results for all three group categories, and the accuracy of the CL participants that reported all group category patterns routinely was comparable to the accuracy of the IVD group. Based on this observation, it is likely that a significant majority of participants that report all three group categories developed competencies for the more challenging (expert-level) patterns. However, although automation of ANA reading holds the promise of improved consistency and accuracy of ANA pattern recognition, automation-assisted reading in CL participants was not associated with a statistically significant improvement in accuracy.

The ICAP guidance is recognized as a potential roadmap towards the harmonization and standardization of HEp-2 IFA nomenclature [31, 32]. It is understood by its members and opinion leaders that this guidance will evolve, taking into consideration practical aspects for its adoption in clinical laboratories; diverse experience, ageing workforce, variability in reagents, microscopy and recent introduction of digital image readers [14, 19, 31]. Along these lines, this investigation is not without limitations. First, the intended responses (traditional nomenclature) for specimens with the cytoplasmic and mitotic patterns were not defined for specific sub-patterns (for example, cytoplasmic speckled or NuMa). This was intentional as it was largely unknown how CL report both patterns. The results obtained from this survey validates the approach, as the minority of laboratories reporting less than 3 main categorical patterns do report mitotic patterns considered expert-level on the ICAP classification tree [www.anapatterns.org, 12]. Second, the intended responses were monospecific and did not take mixed patterns into consideration. A number of participants reported mixed patterns for some of the specimens (data not shown), often with the intended dominant pattern reported together with minor additional pattern variants. Such reports were considered appropriate and in accordance for reporting patient results with more than one pattern [2]. Third, the survey included a limited number of participating CL including those with a significant interest and experience in ANA testing, which may not reflect the experience of a wider spectrum of international CL. Finally, some of the participants, particularly those in CL group, may have limited familiarity with the ICAP nomenclature, despite being associated with experienced laboratories.

The data presented confirm that standardization of reporting has not been achieved in performance of non-traditional HEp-2 patterns even by experienced and interested laboratories. This suggests the need and opportunities for further training and consensus-building. Using the ICAP nomenclature may have benefits for some sub-patterns and assigning competencies, notably for the mitotic and cytoplasmic main categorical groups and our data clearly demonstrate that recognition of the pattern associated with antibodies to topoisomerase is linked to familiarity with ICAP patterns. Furthermore, our data confirm previous observations that differences in the HEp-2 cell substrate can contribute to inconsistency in ANA sub-patterns interpretation and reporting [22]. Clearly, consistent ICAP sub-pattern reporting by laboratories is most meaningful if patterns are commutable using different sources of HEp-2 reagents. The relatively higher competencies of the IVD participants relative to the CL participants is of interest as some laboratories depend on IVD for training as gleaned from AMLI practice survey [19].

## Conclusion

This study highlights significant competency for all participants in identifying the nuclear main categorical HEp-2 IFA patterns. This observation validates the ICAP competent-level classification for this group except for the anti-topoisomerase I antibody pattern. Our data also demonstrate opportunities for defining competencies and training for CL personnel in recognition of cytoplasmic and mitotic patterns.

## Supplementary information

**Additional File 1:** Participating clinical laboratories and in vitro diagnostic manufacturers.

**Additional File 2:** Accuracy of HEp-2 IFA pattern reporting based on type of nomenclature.

## Data Availability

Materials use in the survey was obatined from the Plasma Services Group Inc. (PSG), Huntington Valley, PA, USA. All data from the survey are in the possession AET and MHW.

## References

[1] Meroni PL, Schur PH (2010). ANA screening: an old test with new recommendations. Ann Rheum Dis.

[2] Agmon-Levin N, Damoiseaux J, Kallenberg C, Sack U, Witte T, Herold M (2014). International recommendations for the assessment of autoantibodies to cellular antigens referred to as anti-nuclear antibodies. Ann Rheum Dis.

[3] Pisetsky DS (2017). Antinuclear antibody testing - misunderstood or misbegotten?. Nat Rev Rheumatol.

[4] Damoiseaux J, Andrade LEC, Carballo OG, Conrad K, Francescantonio PL, Fritzler MJ (2019). Clinical relevance of HEp-2 indirect immunofluorescent patterns: the International Consensus on ANA patterns (ICAP) perspective. Ann Rheum Dis.

[5] Emlen W, O’Neill L (1997). Clinical significance of antinuclear antibodies: comparison of detection with immunofluorescence and enzyme-linked immunosorbent assays. Arthritis Rheum.

[6] Homburger HA, Cahen YD, Griffiths J, Jacob GL (1998). Detection of antinuclear antibodies: comparative evaluation of enzyme immunoassay and indirect immunofluorescence methods. Arch Pathol Lab Med.

[7] Tan EM, Smolen JS, McDougal JS, Butcher BT, Conn D, Dawkins R, et al. A critical evaluation of enzyme immunoassays for detection of antinuclear autoantibodies of defined specificities. I. Precision, sensitivity, and specificity. Arthritis Rheum 1999;42:455–64. 10.1002/1529-0131(199904)42:3<455::AID-ANR10>3.0.CO;2-3 10088768

[8] Tonuttia E, Bassetti D, Piazza A, Visentini D, Poletto M, Bassetto F (2004). Diagnostic accuracy of ELISA methods as an alternative screening test to indirect immunofluorescence for the detection of antinuclear antibodies. Evaluation of five commercial kits. Autoimmunity.

[9] Choi MY, Cui J, Costenbader K, Rydzewski D, Bernhard L, Schur P (2020). Different indirect immunofluorescence ANA substrate performance in a diagnostic setting of patients with SLE and related disorders: retrospective review and analysis. Lupus Sci Med..

[10] Copple SS, Sawitzke AD, Wilson AM, Tebo AE, Hill HR (2011). Enzyme-linked immunosorbent assay screening then indirect immunofluorescence confirmation of antinuclear antibodies: a statistical analysis. Am J Clin Pathol.

[11] Olsen NJ, Choi MY, Fritzler MJ (2017). Emerging technologies in autoantibody testing for rheumatic diseases. Arthritis Res Ther.

[12] Chan EK, Damoiseaux J, Carballo OG, Conrad K, de Melo Cruvinel W, Francescantonio PL (2015). Report of the First International Consensus on Standardized Nomenclature of Antinuclear Antibody HEp-2 Cell Patterns 2014-2015. Front Immunol.

[13] Chan EK, Damoiseaux J, de Melo Cruvinel W, Carballo OG, Conrad K, Francescantonio PL (2016). Report on the second International Consensus on ANA Pattern (ICAP) workshop in Dresden 2015. Lupus.

[14] Damoiseaux J, von Mühlen CA, Garcia-De La Torre I, Carballo OG, de Melo Cruvinel W, Francescantonio PL, Fritzler MJ, et al. International consensus on ANA patterns (ICAP): the bumpy road towards a consensus on reporting ANA results. Auto Immun Highlights 2016;7:1. 10.1007/s13317-016-0075-0 PMC4733811 26831867

[15] Hoffman IE, Peene I, Veys EM, De Keyser F (2002). Detection of specific antinuclear reactivities in patients with negative anti-nuclear antibody immunofluorescence screening tests. Clin Chem.

[16] von Mühlen CA, Tan EM (1995). Autoantibodies in the diagnosis of systemic rheumatic diseases. Semin Arthritis Rheum.

[17] Wiik AS (2006). Guidelines for Antinuclear Antibody Testing. EJIFCC.

[18] Herold M, Klotz W, Andrade LEC, Conrad K, Cruvinel WM, Damoiseaux J (2018). International Consensus on Antinuclear Antibody Patterns: defining negative results and reporting unidentified patterns. Clin Chem Lab Med.

[19] Peterson LK, Tebo AE, Wener MH, Copple SS, Fritzler MJ. Assessment of antinuclear antibodies by indirect immunofluorescence assay: report from a survey by the American Association of Medical Laboratory Immunologists. Clin Chem Lab Med. 2020 Apr 8. [Epub ahead of print]. 10.1515/cclm-2019-1262 32271157

[20] Meroni PL, Borghi MO (2018). Diagnostic laboratory tests for systemic autoimmune rheumatic diseases: unmet needs towards harmonization. Clin Chem Lab Med.

[21] Bogaert L, Van den Bremt S, Schouwers S, Bossuyt X, Van Hoovels L (2019). Harmonizing by reducing inter-run variability: performance evaluation of a quality assurance program for antinuclear antibody detection by indirect immunofluorescence. Clin Chem Lab Med.

[22] Andrade LEC, Klotz W, Herold M, Conrad K, Rönnelid J, Fritzler MJ, von Mühlen CA, Satoh M, Damoiseaux J, de Melo Cruvinel W, Chan EKL; Executive Committee of ICAP. International consensus on antinuclear antibody patterns: definition of the AC-29 pattern associated with antibodies to DNA topoisomerase I. Clin Chem Lab Med. 2018;56(10):1783-1788. 10.1515/cclm-2018-0188 29813025

[23] Dellavance A, Gallindo C, Soares MG, Silva NP, Mortara RA, Andrade LE (2009). Redefining the Scl-70 indirect immunofluorescence pattern: autoantibodies to DNA topoisomerase I yield a specific immunofluorescence pattern. Rheumatology.

[24] Turan Faraşat V, Ecemiş T, Doğan Y (2019). A Multicenter Analysis of Subjectivity of Indirect Immunofluorescence Test in Antinuclear Antibody Screening. Arch Rheumatol.

[25] Tan EM, Feltkamp TE, Smolen JS (1997). Range of antinuclear antibodies in “healthy” individuals. Arthritis Rheum.

[26] Egner W (2000). The use of laboratory tests in the diagnosis of SLE. J Clin Pathol.

[27] Sack U, Conrad K, Csernok E (2009). Autoantibody detection using indirect immunofluorescence on HEp-2 cells. Ann N Y Acad Sci.

[28] Banhuk FW, Pahim BC, Jorge AS, Menolli RA (2018). Relationships among Antibodies against Extractable Nuclear Antigens, Antinuclear Antibodies, and Autoimmune Diseases in a Brazilian Public Hospital. Autoimmune Dis.

[29] Tanaka N, Muro Y, Sugiura K, Tomita Y (2008). Anti-SS-A/Ro antibody determination by indirect immunofluorescence and comparison of different methods of anti-nuclear antibody screening: evaluation of the utility of HEp-2 cells transfected with the 60 kDa SS-A/Ro as a substrate. Mod Rheumatol.

[30] Betancur JF, Londoño A, Estrada VE (2018). Uncommon patterns of antinuclear antibodies recognizing mitotic spindle apparatus antigens and clinical associations. Medicine (Baltimore)..

[31] Tebo AE (2017). Recent approaches to optimize laboratory assessment of antinuclear antibodies. Clin Vaccine Immunol.

[32] Damoiseaux J (2020). The perspective on standardisation and harmonisation: the viewpoint of the EASI president. Auto Immun Highlights.

