# Supplementary material for: The antinuclear antibody HEp-2 indirect immunofluorescence assay: a survey of laboratory performance, pattern recognition and interpretation
Source: Auto Immun Highlights. 2021 Feb 27;12(1):4. doi: 10.1186/s13317-020-00146-w (PMC7916270; doi:10.1186/s13317-020-00146-w)
Supplement: Supplementary file 1 — Additional File 1: Participating clinical laboratories and in vitro diagnostic manufacturers. [file 13317_2020_146_MOESM1_ESM.pdf]

Additional File 1. Participating clinical laboratories and *in vitro* diagnostic manufacturers

Participant Group	Name of Rater (Participant), City and Country
Clinical Laboratories (CL)	Ascension, Tulsa, Oklahoma, USA
	LabCorp, Dublin, Ohio, USA
	Hospital for Sick Children, Toronto, Ontario, Canada
	Mayo Clinic Laboratory, Rochester, Minnesota, USA
	ARUP Laboratories, Salt Lake City, Utah, USA
	Toronto General Hospital, Toronto, Ontario, Canada
	Tufts Medical Center, Boston, Massachusetts, USA
	Lexington Medical Laboratories, West Columbia, South Carolina, USA
	Exsera BioLabs, Denver, Colorado, USA
	Mitogen Advanced Diagnostics Laboratories, Calgary, Alberta, Canada
	University of Washington Med Ctr, Seattle, Washington, USA
	KSL Diagnostics, Inc, Buffalo, New York, USA
	Beutner Laboratories, Buffalo, New York, USA
	Virginia Mason Medical Center, Seattle, Washington, USA
	Johns Hopkins University, Baltimore, Maryland, USA
	UNC Hospitals, Chapel Hill, North Carolina, USA
In vitro Diagnostics Manufacturers (IVD)	AESKU Diagnostics, Wendelsheim, Germany
	Bio-Rad, Benicia, California, USA
	Euroimmun, Mountain Lakes, New Jersey, USA
	Immuno Concepts, California, USA
	Inova, San Diego, California, USA
	Scimedx, Dover, New Jersey, USA
	Thermo Fisher, Freiburg, Germany
	Zeus Scientific, Branchburg, New Jersey, USA
