# Supplementary material for: The antinuclear antibody HEp-2 indirect immunofluorescence assay: a survey of laboratory performance, pattern recognition and interpretation
Source: Auto Immun Highlights. 2021 Feb 27;12(1):4. doi: 10.1186/s13317-020-00146-w (PMC7916270; doi:10.1186/s13317-020-00146-w)
Supplement: Supplementary file 2 — Additional File 2: Accuracy of HEp-2 IFA pattern reporting based on type of nomenclature. [file 13317_2020_146_MOESM2_ESM.pdf]

Additional File 2. Accuracy of HEp-2 IFA Pattern Classification based on Type Classification Nomenclatures

A. Accuracy of Classification based on HEp-2 IFA Group Category

Organization Type	Observations	Accuracy (%)	95% CI	p-value
CL	192	91	86-94	0.07
IVD	96	97	91-99	
Overall	276	93	89-96	
CL*	93	94	89-99	0.28
IVD	96	97	93-100	
CL**	87	89	82-95	0.04
CL* and IVD	189	95	92-98	

HEp-2 IFA patterns were evaluated by the two organization types: *in vitro* diagnostics manufacturers (IVD) and clinical laboratories (CL). IFA: indirect immunofluorescence assay, CL: all laboratories, CL*: laboratories that report all 3 group categories, and CL**: laboratories that do not report all 3 group categories.

B. Accuracy of HEp-2 IFA Pattern Classification based on Traditional and ICAP Nomenclatures

Organization Type	Nomenclature		Overall (%)
	Traditional (%)	ICAP (%)	
CL	83 (78-88)	66 (58-73)	75 (70-80)
IVD	93 (91-97)	86 (80-92)	90 (85-93)
Overall	87 (82-90)	74 (68-79)	81 (77-84)

The table shows the accuracy according to two different groups of raters (CL: clinical laboratories, IVD: *in vitro* diagnostics manufacturers), and two nomenclature systems (traditional, and ICAP: **I**nternational **C**onsensus on **A**ntinuclear Antibody **P**atterns); IFA, indirect immunofluorescence assay.
